# Oral Acid Load Down-Regulates Fibroblast Growth Factor 23

**DOI:** 10.3390/nu14051041

**Published:** 2022-02-28

**Authors:** Angela Vidal, Carmen Pineda, Ana I. Raya, Rafael Rios, Azahara Espartero, Juan R. Muñoz-Castañeda, Mariano Rodriguez, Escolastico Aguilera-Tejero, Ignacio Lopez

**Affiliations:** 1 Department of Animal Medicine and Surgery, University of Cordoba, Campus Universitario Rabanales, 14014 Cordoba, Spain; v92vicaa@uco.es (A.V.); cpimart@hotmail.com (C.P.); ana.raya@uco.es (A.I.R.); rafariosvaro@me.com (R.R.); azaharaespartero@hotmail.com (A.E.); l02lovii@uco.es (I.L.); 2 Maimonides Biomedical Research Institute of Cordoba (IMIBIC), Reina Sofia University Hospital, University of Cordoba, 14004 Cordoba, Spain; juanr.munoz.exts@juntadeandalucia.es (J.R.M.-C.); marianorodriguezportillo@gmail.com (M.R.)

**Keywords:** acid intake, FGF23, PTH, kidney

## Abstract

Increased dietary acid load has a negative impact on health, particularly when renal function is compromised. Fibroblast growth factor 23 (FGF23) is a bone-derived hormone that is elevated during renal failure. The relationship between metabolic acidosis and FGF23 remains unclear. To investigate the effect of dietary acid load on circulating levels of FGF23, rats with normal renal function and with a graded reduction in renal mass (1/2 Nx and 5/6 Nx) received oral NH_4_Cl for 1 month. Acid intake resulted in a consistent decrease of plasma FGF23 concentrations in all study groups when compared with their non-acidotic control: 239.3 ± 13.5 vs. 295.0 ± 15.8 pg/mL (intact), 346.4 ± 19.7 vs. 522.6 ± 29.3 pg/mL (1/2 Nx) and 988.0 ± 125.5 vs. 2549.4 ± 469.7 pg/mL (5/6 Nx). Acidosis also decreased plasma PTH in all groups, 96.5 ± 22.3 vs. 107.3 ± 19.1 pg/mL, 113.1 ± 17.3 vs. 185.8 ± 22.2 pg/mL and 504.9 ± 75.7 vs. 1255.4 ± 181.1 pg/mL. FGF23 showed a strong positive correlation with PTH (r = 0.877, *p* < 0.0001) and further studies demonstrated that acidosis did not influence plasma FGF23 concentrations in parathyroidectomized rats, 190.0 ± 31.6 vs. 215 ± 25.6 pg/mL. In conclusion, plasma concentrations of FGF23 are consistently decreased in rats with metabolic acidosis secondary to increased acid intake, both in animals with intact renal function and with decreased renal function. The in vivo effect of metabolic acidosis on FGF23 appears to be related to the simultaneous decrease in PTH.

## 1. Introduction

Nutrient intake plays a major role in the maintenance of acid-base homeostasis [1]. An excessive dietary acid load may lead to metabolic acidosis, particularly in individuals with chronic kidney disease (CKD) because the failing kidney is not able to produce the amount of bicarbonate required to buffer acid production resulting from diet and metabolism [2,3,4]. Chronic metabolic acidosis is thought to represent a major factor in the progression of CKD [5] and is generally associated with higher mortality risk [6].

Fibroblast growth factor 23 (FGF23) is a hormone secreted by osteocytes/osteoblasts that is involved in the regulation of mineral metabolism [7]. The main target organ for FGF23 is the kidney, where FGF23 increases urinary excretion of phosphate (P) and decreases the synthesis of calcitriol [8].

Synthesis and secretion of FGF23 are regulated mainly by dietary P. In fact, an increased P intake is thought to be the most important stimulus for FGF23 secretion [9,10]. In addition, FGF23 production is under hormonal control by calcitriol [11,12] and by parathyroid hormone (PTH), which stimulates FGF23 secretion [13]. Moreover, inflammation [14], iron deficiency [15] and high caloric intake [16] have been reported to increase FGF23 production. 

Elevated levels of FGF23 are usually found in patients with renal failure, due to P retention and hyperphosphatemia, and FGF23 has been reported as a risk factor of cardiovascular mortality both in patients with CKD [17] and in healthy individuals [18,19].

The connection between FGF23 and metabolic acidosis is unclear. How acidosis affects FGF23 production should be known because it may influence dietary recommendations for the general population and, particularly, for persons with reduced renal function. In vitro studies have shown that acidosis stimulates FGF23 production by neonatal mouse bone [20,21]. However, to our knowledge, in vivo studies specifically designed to investigate the effect of metabolic acidosis on FGF23 have not been conducted. Interestingly, the few collateral data that have been reported from in vivo studies, that were originally designed for other purposes, do not support a stimulatory effect of acidosis on FGF23 [22,23,24]. Thus, it is necessary to clarify the relationship between metabolic acidosis and increased FGF23 concentrations.

The aim of this work was to test the hypothesis that, as previously shown in vitro, metabolic acidosis also up-regulates circulating levels of FGF23 in vivo. To this purpose, we investigated the influence of increased acid intake (in the form of NH_4_Cl supplied with drinking water) on FGF23 in healthy rats and in rats with several degrees of impairment in renal function. In addition, in vitro studies were conducted in UMR 106 cells cultured at different pH.

## 2. Materials and Methods

### 2.1. Ethics

Experimental protocols were reviewed and approved by the Ethics Committee for Animal Research of the University of Cordoba and by Junta de Andalucia (Spain) (Ethical Code Number 30/10/2017/148, dated 8 November 2017). All the studies were carried out in accordance with the approved guidelines. 

### 2.2. In Vivo Experiments

#### 2.2.1. Animals and Diets

Two months old female Wistar rats, provided by the Animal Housing Facilities of the University of Cordoba (Cordoba, Spain), were used in the studies. 

Dietary modification, including changes in P and fat content, was used as a part of the treatment. Three diets were used in the experiments: a standard diet (Altromin C 1090-10, AltrominSpezialfutter GmbH, Lage, Germany) with either a normal (0.6%) or high (1.2%) P concentration, and a diet with high-fat (HF) content and 0.6% P concentration (Altromin C 1090-60, AltrominSpezialfutter GmbH, Germany). These diets contained normal amounts of calcium (Ca) and vitamin D: 0.6% of Ca and 500 IU/g of vitamin D. Daily food intake was recorded for each rat by averaging the amount of food eaten every week.

#### 2.2.2. Generation of Kidney Disease

Renal function was reduced by partial removal of renal mass and by dietary changes (increasing fat and P intake). For ablation of renal mass, two nephrectomy (Nx) procedures were carried out: 1/2 Nx and 5/6 Nx. Before performing surgery, rats were anesthetized using inhaled isoflurane (Isovet, Braun, Barcelona, Spain). For 1/2 Nx, the right kidney was exposed and removed. For 5/6 Nx, it was performed in two steps. In the first step, the left kidney was exposed, and the two poles (2/3 of renal mass) were ablated. After 1 week of recovery, in the second step, the animal was reanesthetized and the right kidney was excised, as described above. Fentanyl, 0.2 mg/kg, ip (Fentanest, Kern Pharma, Barcelona, Spain) was used as an analgesic agent. Rats subjected to 1/2 Nx were fed an HF diet to accelerate the deterioration of renal function induced by heminephrectomy. 

#### 2.2.3. Parathyroidectomy

A selective parathyroidectomy (PTX) was performed, as previously reported, with the aid of a dissecting microscope [13]. Briefly, with the rat under general anesthesia (inhaled isofluorane), the skin on the ventral part of the neck was incised, the thyroid was exposed, and the parathyroid glands were identified, dissected and ablated. Hemorrhage was prevented by electrocautery. 

#### 2.2.4. Induction of Acidosis

Metabolic acidosis was induced in rats with intact renal function and in 1/2 Nx rats by oral administration of a NH_4_Cl solution, 1% NH_4_Cl (Scharlau, Scharlab, Barcelona, Spain) dissolved in water, instead of normal drinking water. In 5/6 Nx rats, the NH_4_Cl concentration was reduced to 0.75%. Control (non-acidotic) rats received tap water. In all groups, 0.1% sodium saccharin (Scharlau, Scharlab, Barcelona, Spain) was added to drinking water to improve its palatability. 

#### 2.2.5. Experimental Design

(a) Acidosis with intact parathyroid function

Rats were randomly allotted to 6 groups (*n* = 9 per group) using specific software (Research Randomizer). The number of rats per group was calculated based on the dispersion observed when measuring the parameters under investigation in previous studies by our laboratory, and assuming a 95% confidence level. Three groups (Groups 1, 3 and 5) included rats with normal acid-base balance and 3 groups (Groups 2, 4, and 6) included rats with acidosis (Figure S1):

Group 1 (Control), rats with an intact renal function that were fed a standard diet (0.6% P).

Group 2 (Control+Acid) was similar to Group 1, but the rats received NH_4_Cl to induce metabolic acidosis.

Group 3 (1/2 Nx), 1/2 Nx rats fed HF diet.

Group 4 (1/2 Nx+Acid) was similar to Group 3, with the addition of NH_4_Cl to induce chronic metabolic acidosis.

Group 5 (5/6 Nx), rats with 5/6 Nx were fed a standard diet with high P (1.2% P).

Group 6 (5/6 Nx+Acid) was similar to Group 5 plus NH_4_Cl to induce chronic metabolic acidosis.

To confirm an even distribution of rats between the different experimental groups, rats were weighed before commencing the experiments. No significant difference in the body weight of the study groups was found (Group 1 = 219.7 ± 5.2 g, Group 2 = 226.4 ± 5.8 g, Group 3 = 221.3 ± 2.3 g, Group 4 = 216.6 ± 2.2 g, Group 5 = 226.7 ± 3.0 g and Group 6 = 227.5 ± 2.6 g).

Rats received the treatments for 30 days and at the end of the experiments were sacrificed by exsanguination under general anesthesia (inhaled isoflurane) to obtain blood samples (from the abdominal aorta). For twenty-four hours, urine samples were collected for the 3 days prior to sacrifice by placing the rats in metabolic cages.

(b) Acidosis with parathyroidectomy

Rats with intact renal function fed a standard diet (Altromin C 1090-10, AltrominSpezialfutter GmbH, Lage, Germany) were subjected to PTX, as described above. Twenty-four hours after parathyroidectomy, rats were allotted to two groups: PTX (*n* = 6) and PTX+Acid (*n* = 8). PTX (non-acidotic) rats received tap water, while PTX+Acid received a 1% NH_4_Cl solution instead of drinking water, as described above. Both groups of rats were euthanized after 30 days of treatment. Blood and urine collections were performed as described above.

#### 2.2.6. Blood and Urine Chemistries

Arterial blood gas analysis and ionized calcium (Ca^2+^) measurements were performed immediately after blood collection using selective electrodes (RapidLab, Siemens Healthcare GmbH, Germany). Afterwards, plasma was separated by centrifugation and stored at –20 °C until assayed. Plasma concentrations of P, creatinine and urea, and urine Ca (total Ca) and P were measured by spectrophotometry (Biosystems SA, Barcelona, Spain). Urine pH was measured with pH indicator strips (Scharlau, Scharlab SL, Barcelona, Spain). ELISA tests were used to quantify intact FGF23, iFGF23 (Kainos Laboratories, Tokyo, Japan), carboxy-terminal FGF23, cFGF23 (Immutopics Inc., Quidel Corporation, OH, USA) and PTH (Immutopics Inc., Quidel Corporation, OH, USA). Radioimmunoassay (Immunodiagnostic Systems Ltd., Boldon, UK) was used in plasma samples to determine 1,25-dihydroxyvitamin D (calcitriol). 

### 2.3. In Vitro Experiments

#### 2.3.1. Cell Culture

Rat osteosarcoma cells UMR 106 (ATCC, Manassas, VA, USA) were cultured in Dulbecco’s modified Eagle’s medium (DMEM) (Sigma-Aldrich, St. Louis, MO, USA) supplemented with 10% fetal bovine serum (FBS) (Biowest, Riverside, MO, USA). Cells were seeded in 6 wells plates with 10,000 cells/cm^2^ and maintained in DMEM up to 90% confluence. FGF23 production was stimulated by adding calcitriol (10^−8^ M) (Kern pharma, Barcelona, Spain), as previously reported [25]. Two groups of experiments were performed to study the effect of acidosis on FGF23 production: short-term and longer-term experiments. Short-term experiments were carried out in cells that had grown in a medium with normal (7.4) pH and were briefly exposed (for 24 h) to a low (7.2) pH medium. The pH of the culture medium was reduced by adding HCl. In longer-term experiments, cells were incubated until they reached confluence (for 6 days) in either DMEM with normal pH (7.4) or DMEM with low pH (7.2).

#### 2.3.2. RNA Extraction and Real-Time Reverse Transcription-Polymerase Chain Reaction (RT-PCR)

Total RNA was isolated using the TRIzol reagent protocol (Invitrogen, Thermo Fisher Scientific, Walthan, MA, USA). The sequence of primers used for RT-PCR is shown in Table S1. Quantification was done using the QuantiTect SYBR Green RT-PCR kit (Qiagen GmbH, Hilden, Germany) for 50 ng of RNA and 1 µL of primer. The mRNA expression was analyzed in the Light Cycler thermal cycler system (Roche Diagnostics, Indianapolis, IN, USA) and the relative expression of the target genes was determined using the 2^−ΔΔCt^ method.

### 2.4. Statistical Analysis

Statistical analysis was conducted with the software Prism/GraphPad v6. Values were reported as mean ± standard error (SE). To compare the difference of the means of 3 or more groups, ANOVA, followed by the Fisher LSD test as a post-hoc procedure was used. When only 2 groups were involved, an intergroup comparison was performed by t-tests. Pearson correlation coefficients were used to estimate and assess the strength of associations. A *p* < 0.05 was considered significant.

## 3. Results

### 3.1. In Vivo Studies

#### 3.1.1. Renal Function

Plasma creatinine and urea concentrations in the study groups are shown in Table 1. Acidosis tended to decrease plasma creatinine, but significant differences with the non-acidotic controls were only observed in 5/6 Nx rats.

#### 3.1.2. Acid-Base Balance

**Figure 1:**
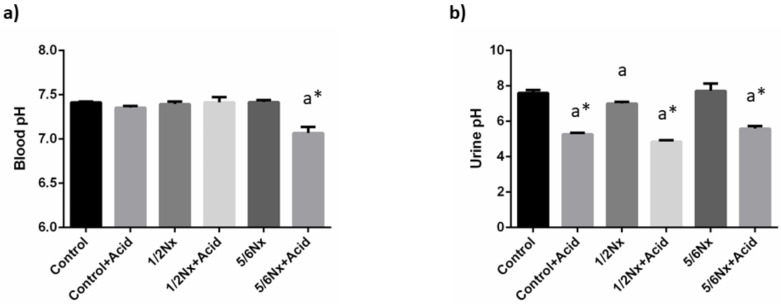
Acid-base parameters. (**a**) Blood pH, and (**b**) urine pH in rats (*n* = 9 per group) with normal (control) and reduced (1/2 Nx and 5/6 Nx) renal function without and with (+Acid) metabolic acidosis. ^a^ *p* < 0.05 vs. control; * *p* < 0.05 vs. its non-acidotic counterpart.

As intended, mild metabolic acidosis was achieved in rats with normal renal function and in 1/2 Nx rats. Thus, a decrease in blood pH was only observed in 5/6 Nx rats, 7.07 ± 0.07 vs. 7.42 ± 0.02. However, a significant reduction in urine pH was detected in all acidotic groups (Figure 1). Changes in plasma bicarbonate were only apparent in acidotic 5/6 Nx rats, while the anion gap was increased in the non-acidotic 5/6 Nx group (Table 2). 

#### 3.1.3. Mineral Metabolism

**Figure 2:**
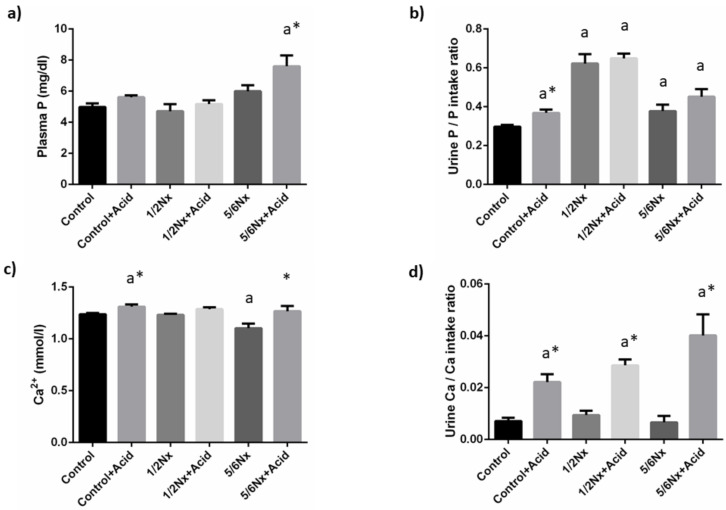
Plasma concentrations and urinary excretion of phosphate and calcium. (**a**) Plasma phosphate, P, (**b**) urinary excretion of P, (**c**) plasma ionized calcium, Ca^2+^, and (**d**) urinary excretion of Ca in rats (*n* = 9 per group) with normal (control) and reduced (1/2 Nx and 5/6 Nx) renal function without and with (+Acid) metabolic acidosis. ^a^ *p* < 0.05 vs. control. * *p* < 0.05 vs. its non-acidotic counterpart.

Plasma P concentrations tended to increase with the reduction in renal function and acidosis further increased plasma P in 5/6 Nx rats, 7.6 ± 0.7 vs. 6.0 ± 0.4 mg/dL. Since acidotic rats decreased food intake and, consequently P intake, when compared with non-acidotic controls (Table S2), urinary excretion of P was expressed as the ratio daily urinary P excretion (mg/day)/daily P ingestion (mg/day). Urinary excretion of P increased in rats with reduced renal function, particularly in 1/2 Nx rats. Acidosis resulted in a further increase in urinary P excretion in all groups and significant differences were observed in rats with intact renal function, 0.37 ± 0.02 vs. 0.30 ± 0.01 (Figure 2).

Blood Ca^2+^ concentrations did not change in 1/2 Nx rats but were significantly reduced in 5/6 Nx rats, 1.10 ± 0.04 vs. 1.24 ± 0.01 mmol/L in controls with normal renal function. Acidosis resulted in increases in Ca^2+^ that were significant both in controls, 1.31 ± 0.02 mmol/L and in 5/6 Nx, 1.27 ± 0.05 mmol/L. Urinary excretion of Ca was consistently increased in all acidosis groups (Figure 2).

**Figure 3:**
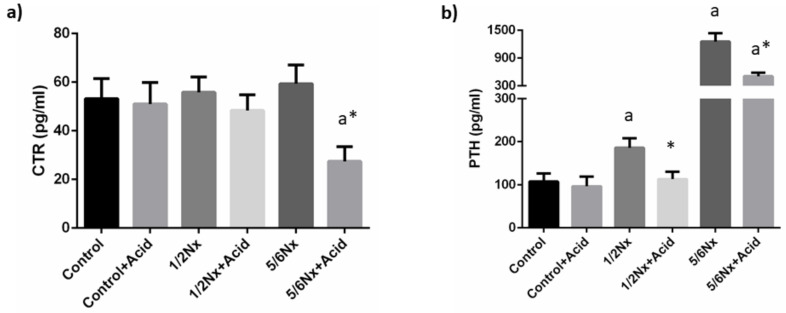
Plasma concentrations of calcitriol and parathyroid hormone. (**a**) Calcitriol (CTR) and (**b**) parathyroid hormone (PTH) in rats (*n* = 9 per group) with normal (control) and reduced (1/2 Nx and 5/6 Nx) renal function without and with (+Acid) metabolic acidosis. ^a^ *p* < 0.05 vs. control; * *p* < 0.05 vs. its non-acidotic counterpart.

Plasma calcitriol concentrations were not substantially altered by the reduction in renal function. Acidosis tended to decrease plasma calcitriol and significant differences were recorded in 5/6 Nx rats, 27.4 ± 6.1 vs. 59.4 ± 7.7 pg/mL (Figure 3).

Circulating PTH concentrations increased progressively with the deterioration of renal function, from 107.3 ± 19.1 in rats with normal renal function to 1255.4 ± 181.1 pg/mL in 5/6 Nx rats. Acidosis resulted in a decrease in plasma PTH in all groups, 96.5 ± 22.3 vs. 107.3 ± 19.1 pg/mL, 113.1 ± 17.3 vs. 185.8 ±22.2 pg/mL and 504.9 ± 75.7 vs. 1255.4 ± 181.1 pg/mL, although significant differences were only observed in rats with reduced renal function (Figure 3).

**Figure 4:**
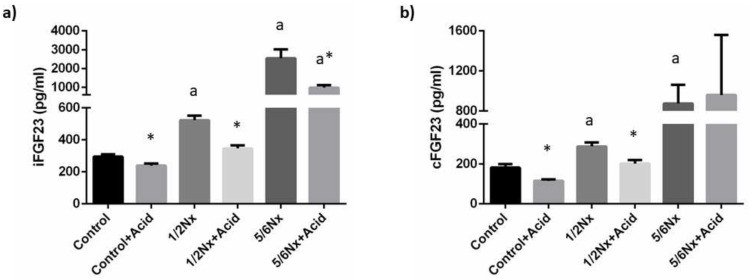
Plasma concentrations of fibroblast growth factor 23 (FGF23). (**a**) Intact FGF23, iFGF23, and (**b**) carboxy-terminal FGF23, cFGF23, in rats (*n* = 9 per group) with normal (control) and reduced (1/2 Nx and 5/6 Nx) renal function without and with (+Acid) metabolic acidosis. ^a^ *p* < 0.05 vs. control; * *p* < 0.05 vs. its non-acidotic counterpart.

Plasma concentrations of iFGF23 increased progressively in the study groups as a reflection of the decline in renal function, from 295.0 ± 16.8 pg/mL, in rats with intact renal function to 2549.4 ± 469.7 pg/mL in 5/6 Nx rats. Acidosis resulted in a consistent decrease of iFGF23 in all study groups when compared with their non-acidotic control: 239.3 ± 13.5 vs. 295.0 ± 15.8 pg/mL (intact renal function), 346.4 ± 19.7 vs. 522.6 ± 29.3 pg/mL (1/2 Nx) and 988.0 ± 125.5 vs. 2549.4 ± 469.7 pg/mL (5/6 Nx) (Figure 4). Circulating levels of cFGF23 were increased with deteriorated renal function and were also significantly reduced in acidotic rats with normal renal function, 115.2 ± 7.3 vs. 181.0 ± 17.5 pg/mL, and in 1/2 Nx rats, 201.3 ± 17.7 vs. 287.2 ± 19.9 pg/mL but not in 5/6 Nx rats, 959.4 ± 600.5 vs. 874.7 ±187.0 pg/mL (Figure 4). However, the 5/6 Nx results were influenced by an outlier in the acidotic group that had an extremely high cFGF23 concentration (2751.5 pg/mL). When the data from this rat was not included in the statistical analysis, a decrease in cFGF23 was also observed in acidotic 5/6 Nx rats, 362.0 ± 85.91 vs. 874.7 ± 187.0 (*p* = 0.142).

**Figure 5:**
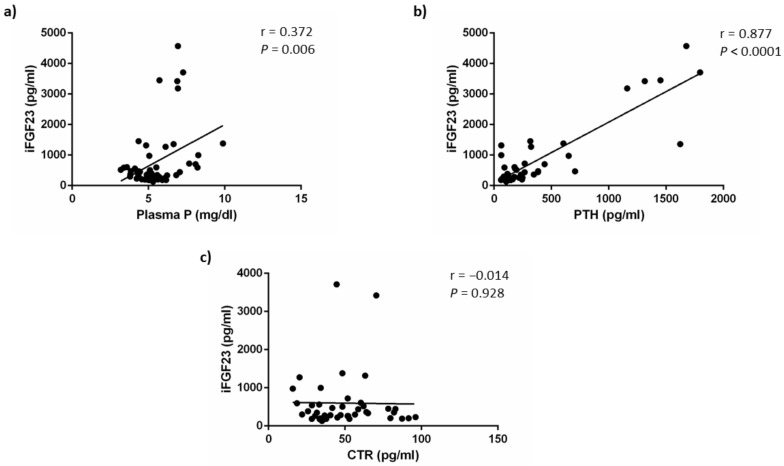
iFGF23 correlations. Correlation between plasma concentrations of intact fibroblast growth factor 23 (iFGF23) and: (**a**) plasma phosphate, P, (**b**) plasma parathyroid hormone, PTH and (**c**) plasma calcitriol, CTR, concentrations.

As shown in Figure 5, iFGF23 concentrations were weakly correlated with plasma P concentrations (r = 0.372, *p* = 0.006) but this direct correlation was a likely consequence of the simultaneous increases of P and FGF23 secondary to a deterioration of renal function and may not reflect the effect of acidosis on FGF23. By contrast, iFGF23 showed a strong positive correlation with plasma PTH concentrations (r = 0.877, *p* < 0.0001). No correlation was found between iFGF23 and calcitriol concentrations (r = −0.014, *p* = 0.928). The correlation study yielded similar results when it was carried out using cFGF23 data (Figure S2).

To further explore the role of PTH in the acidosis-induced decrease in FGF23, in vivo experiments were repeated in a subset of parathyroidectomized (PTX) rats with normal renal function. In these rats, acid ingestion resulted in significant decreases in plasma pH, 7.27 ± 0.02 vs. 7.40 ± 0.01, and bicarbonate, 16.7 ± 1.0 vs. 24.6 ± 0.5 mmol/L. However, iFGF23 did not change in PTX rats subjected to acidosis, 190.0 ± 31.6 vs. 215.9 ± 25.6 pg/mL (Table 3). 

### 3.2. In Vitro Studies

UMR 106 cells increased FGF23 mRNA expression (arbitrary units vs. tbp) when exposed to acidic medium both short-term (24 h), 1.23 ± 0.07 vs. 1.01 ± 0.03 (*p* = 0.004) and longer-term (6 days) 1.21 ± 0.08 vs. 1.01 ± 0.04 (*p* = 0.032).

## 4. Discussion

This study aimed to elucidate the effect of increased acid intake on circulating FGF23 concentrations. Our results demonstrate that metabolic acidosis was associated with lower plasma FGF23 concentrations. This effect was very consistent and reproducible along a scale of renal function (from intact to markedly impaired). The decrease in FGF23 in chronic metabolic acidosis was associated with a simultaneous decrease in PTH and was not observed after PTX. 

Increased levels of FGF23 and metabolic acidosis are detrimental to health, and they contribute to the progression of renal disease and survival [5,17]. Thus, the present study was designed to investigate the effect of metabolic acidosis on plasma FGF23 concentrations in healthy rats and in rats with impaired renal function. The reduction in renal function was graded to simulate clinical situations of CKD stages 2–3 and stage 4. A moderate reduction in renal function was achieved by a heminephrectomy combined with feeding high-fat diets. This model, which has been used previously by our group [26], combines ablation of renal mass and subtle damage to the remnant kidney, which mimics human CKD associated with metabolic syndrome. The 5/6 Nx combined with a high P diet is the most common procedure for the induction of severe kidney disease in rats [27]. Metabolic acidosis was achieved by oral administration of NH_4_Cl, which is also a universal method to produce acidosis in rodents [28]. Many studies of acidosis in rats have induced severe acidosis that may not be translated to patients with CKD. Thus, we aimed to achieve both mild acidosis (in rats with intact renal function and with 1/2 Nx) and severe acidosis (in 5/6 Nx rats).

Previous work has shown that in vitro, metabolic acidosis stimulates FGF23 production by neonatal mouse calvaria cells [20,21]. Our results confirm these findings using a different cell line, UMR 106. A recent study also demonstrates a stimulatory action of weak acids (e.g., lactate) on FGF23 production in vitro, which is attributed to both a pH-mediated effect and an inflammation-mediated effect [29]. Typically, in vitro experiments are of short duration (e.g., 24 h) and this might account for the difference between in vitro and in vivo studies. However, our data demonstrate that the influence of acidosis on FGF23 in vitro is not restricted to short-term experiments (24 h) but it can also be demonstrated in longer-term (6 days) experiments. Taken together, these results would indicate that the direct effect of acidosis on bone is to stimulate FGF23 secretion. It is interesting to point out that acidosis has also been shown to stimulate the production of another phosphaturic hormone, PTH [30].

However, based on our results, metabolic acidosis secondary to increased acid intake resulted in a consistent decrease in plasma FGF23 concentrations. Although to our knowledge, this is the first report documenting a decrease in FGF23 as a consequence of acidosis—there are some data in the literature that point in the same direction.

In a large cross-sectional study involving 980 CKD patients, Khairallah et al. investigated the relationship between circulating FGF23 concentrations and acid-base balance [24]. They did not find an association between FGF23 and either the potential acid load or bicarbonate, however, FGF23 tended to be lower in patients with an increased net acid excretion, although the differences were not significant when data were adjusted for clinical factors (estimated glomerular filtration rate, 24-h urine albumin, diabetes, etc.). It is interesting to note that in this study, cFGF23 was measured and, according to our data, advanced renal disease may tamper the effect of acidosis on cFGF23.

In a study designed to investigate the effect of acidosis on vascular calcification, Leibrock et al. found that NH_4_Cl administration decreased iFGF23 in klotho-deficient mice but not in wild-type mice. These changes were observed only in iFGF23, but not in cFGF23 [22]. 

In CKD patients with mild metabolic acidosis (plasma bicarbonate 20–24 mEq/l) Chen et al. administered increasing doses of oral sodium bicarbonate for 6 weeks. Contrary to their expectations, these authors found an increase in plasma cFGF23 concentrations after bicarbonate treatment and concluded that it was unclear whether the increase in cFGF23 would be related to the alkalosis induced by bicarbonate [23]. 

Given the discrepancy between in vitro and in vivo studies, it is likely that the in vivo effect of acidosis may be related to changes in other parameters of mineral metabolism that regulate FGF23 production by bone cells. The main factors that impact FGF23 synthesis and secretion are P, calcitriol and PTH [9,10,11,12,13].

The most important biological action of FGF23 is to promote phosphaturia, therefore, it seems logical that P should have a predominant effect on FGF23. The effect of acidosis on plasma P is controversial; plasma P has been reported to decrease in short-term studies carried out in healthy individuals [31] and to increase in renal patients with chronic metabolic acidosis [17]. In the course of acidosis, P is extracted from bone [32] and from soft tissues [33]. Moreover, intestinal absorption of P is also increased during acidosis [34]. All these factors are likely responsible for the increase in plasma P detected in our acidotic rats with reduced renal function; however, the elevated plasma P does not explain the decrease in FGF23. An increase in the urinary excretion of P and Ca, which is likely related to mineral extraction from bone [32], was found in acidotic rats. Acidosis has also been reported to increase phosphaturia by down-regulation of Na/P cotransporters Na/Pi2a and Na/Pi2c, in a process that seems dependent on the availability of P [35,36]. However, more recent data have questioned the role of down-regulation of protein expression of Na/P cotransporters and attributes the increased phosphaturia to reduced transporter activity after the interaction of protons with Na/Pi2a and Na/Pi2c [37]. It is interesting to note that acidotic rats had increased phosphaturia even though FGF23 and PTH levels were decreased. Mechanistically, it would make sense to decrease FGF23 secretion in a situation in which phosphaturia is already increased by acidosis. 

Calcitriol also regulates FGF23 synthesis [12] and the stimulatory effect of calcitriol on FGF23 has been demonstrated both in healthy bone and in bone from CKD patients [38]. Metabolic acidosis is known to inhibit CYP27B1, the main enzyme that participates in calcitriol synthesis [39], although the influence of acidosis on circulating levels of vitamin metabolites D is controversial [31]. In our rats with acidosis, there was a tendency to decreased calcitriol, which may have played an additional role in the decrease in FGF23. However, the almost null correlation between calcitriol and FGF23 does not support a major influence of calcitriol on acidosis-induced FGF23 down-regulation.

PTH is another main regulator of FGF23. FGF23 concentrations have been reported to be reduced in parathyroidectomized rats, and PTH supplementation increases plasma FGF23 in a dose-dependent manner [13]. The effect of PTH on FGF23 is partially mediated by calcitriol, but PTH is able to increase FGF23 under conditions in which calcitriol is controlled [13]. Metabolic acidosis has been shown to stimulate PTH secretion [30]; however, this only occurs when plasma ionized calcium levels are clamped. In fact, the increase in ionized calcium secondary to acidosis can revert the changes in PTH [40]. The acidotic rats of the present study showed increased plasma Ca^2+^ concentrations, which were likely due to two mechanisms: (a) acidosis-induced mobilization of Ca from bone; and (b) acidosis-induced increase in the ionized fraction of Ca (by shifting the equilibrium between protein-bound and ionized Ca). In agreement with the increased Ca^2+^, in our study, plasma PTH was consistently decreased in acidotic rats. In addition, an excellent correlation was found between plasma concentrations of PTH and FGF23. Moreover, when PTH was eliminated (in PTX rats), FGF23 did not decrease in response to acidosis. It is also interesting to note that diet-induced acidosis lowers PTH response in experimental models [41]. Thus, it is likely that changes in PTH may play a major role in the decrease in FGF23 observed in acidotic rats. 

**Figure 6:**
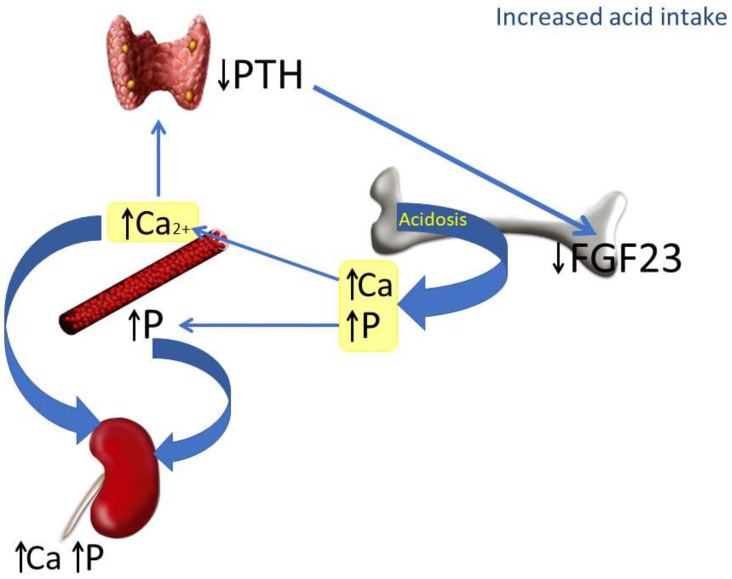
Proposed model integrating the actions of chronic metabolic acidosis on bone, kidney and parathyroid glands. As a consequence of metabolic acidosis, calcium (Ca) and phosphate (P) are extracted from bone. This contributes to elevating calcemia and phosphatemia and increasing urinary excretion of both Ca and P. The increase in plasma Ca^2+^, which is further potentiated by acidosis, inhibits PTH secretion by parathyroid glands and down-regulates FGF23 production by bone cells.

The mechanisms involved in the reduction of FGF23 induced by increased acid intake are depicted in Figure 6. In the context of acidosis, calcium and phosphate are released from bone to buffer the acid load and are abundantly excreted in the urine. The increase in Ca^2+^ inhibits PTH secretion, which in turn down-regulates FGF23. The decrease in FGF23 makes sense from a regulatory perspective: in the first place, because it is not needed to increase phosphaturia (which is already stimulated by acidosis), and also because of its collateral effects on calcitriol and PTH. A decrease in FGF23 will prevent a further decrease in calcitriol concentrations and will also contribute to avoiding increases in PTH, which could be detrimental for a bone tissue already subjected to the decalcifying influence of acidosis. 

Both increased FGF23 and lowered bicarbonate concentrations are considered factors that increase cardiovascular morbidity/mortality [17,42]. In fact, bicarbonate (and other alkali producing foods, like fruits and vegetables) are advocated as therapeutic strategies to reduce cardiovascular risk, particularly in patients with CKD [43]. Since bicarbonate treatment is likely to increase FGF23, it would be interesting to investigate the balance point between acid-base status and FGF23 concentrations that would be more favorable for the cardiovascular system.

In addition to traditional factors related to mineral metabolism and renal disease, there is some evidence that acidosis may influence energy metabolism. Acidosis may impair glycemic control and promote insulin resistance [44]. Insulin has been shown to decrease FGF23 secretion by bone cells [25]. At present, it is unclear whether acidosis-induced insulin resistance may influence the reduction of FGF23 observed in acidotic rats.

This paper has some strengths and also some limitations. The main strength is the use of different animal models which allow the evaluation of the FGF23 response to acidosis in a variety of clinically relevant settings. Limitations include the fact that the results need to be confirmed in humans. The discrepancy between the in vivo and in vitro effect of acidosis on FGF23 secretion needs to be further explored. Although the influence of PTH on the acidosis-induced decrease in FGF23 seems clear, additional factors may also be playing a role in this process.

## 5. Conclusions

In conclusion, in rats with increased acid intake, plasma concentrations of FGF23 are consistently decreased, both in animals with intact renal function and with decreased renal function. The in vivo effect of metabolic acidosis on FGF23 appears to be related to the simultaneous decrease in PTH, which is secondary to an elevation of plasma Ca^2+^ concentration.

## Figures and Tables

**Table 1:** Plasma creatinine and urea concentrations in the six experimental groups at the end of the experiment.

	Creatinine (mg/dL)	Urea (mg/dL)
Control	0.66 ± 0.01	30.2 ± 1.2
Control+Acid	0.63 ± 0.01	27.0 ± 0.8
1/2 Nx	0.80 ± 0.02 ^a^	39.1 ± 1.8 ^a^
1/2 Nx+Acid	0.76 ± 0.01 ^a^	34.1 ± 1.5
5/6 Nx	1.12 ± 0.05 ^a^	70.3 ± 7.7 ^a^
5/6 Nx+Acid	0.94 ± 0.03 ^a^*	82.1 ± 10.1 ^a^

Letters (^a^) indicate significant differences (*p* < 0.05) vs. control group. Asterisks (*) indicate differences vs. its non-acidotic counterpart (*p* < 0.05). Values are means ± SE. Control, rats with intact renal function fed a standard diet. Control+Acid, rats with intact renal function fed a standard diet and induced metabolic acidosis. 1/2 Nx, heminephrectomized rats fed a high-fat diet. 1/2 Nx+Acid, heminephrectomized rats fed a high-fat diet and induced metabolic acidosis. 5/6 Nx, rats subjected to 5/6 nephrectomy fed a high phosphorous diet. 5/6 Nx+Acid, rats subjected to 5/6 nephrectomy fed a high phosphorous diet and induced metabolic acidosis.

**Table 2:** Blood parameters related to acid-base balance in the six experimental groups at the end of the experiment.

	Bicarbonate (mmol/L)	Anion Gap (mmol/L)
Control	25.9 ± 0.5	11.4 ± 0.4
Control+Acid	24.7 ± 0.7	10.6 ± 0.6
1/2 Nx	24.9 ± 0.3	10.9 ± 0.6
1/2 Nx+Acid	23.7 ± 0.6	10.6 ± 0.4
5/6 Nx	24.2 ± 1.1	20.3 ± 2.8 ^a^
5/6 Nx+Acid	12.6 ± 2.9 ^a^*	15.6 ± 1.0 *

Letters (^a^) indicate significant differences (*p* < 0.05) vs. control group. Asterisks (*) indicate differences vs. its non-acidotic counterpart (*p* < 0.05). Values are means ± SE. Control, rats with intact renal function fed a standard diet. Control+Acid, rats with intact renal function fed a standard diet and induced metabolic acidosis. 1/2 Nx, heminephrectomized rats fed a high-fat diet. 1/2 Nx+Acid, heminephrectomized rats fed a high-fat diet and induced metabolic acidosis. 5/6 Nx, rats subjected to 5/6 nephrectomy fed a high phosphorous diet. 5/6 Nx+Acid, rats subjected to 5/6 nephrectomy fed a high phosphorous diet and induced metabolic acidosis.

**Table 3:** Blood parameters related to acid-base balance and plasma intact fibroblast growth factor 23 (iFGF23) concentrations in parathyroidectomized rats.

	pH	Bicarbonate (mmol/L)	iFGF23 (pg/mL)
PTX	7.40 ± 0.01	24.6 ± 0.5	215.9 ± 25.6
PTX+Acid	7.27 ± 0.02 *	16.7 ± 1.0 *	190.0 ± 31.6

Asterisks (*) indicate differences vs. non-acidotic group (*p* < 0.05). Values are means ± SE. PTX, non-acidotic parathyroidectomized rats. PTX+Acid, parathyroidectomized rats with induced metabolic acidosis.

## Data Availability

The datasets generated during and/or analyzed during the current study are available from the corresponding author on reasonable request.

## Supplementary Materials

The following supporting information can be downloaded at: www.mdpi.com/article/10.3390/nu14051041/s1, Figure S1: Experimental design. Figure S2: Correlation between plasma concentrations of carboxy-terminal fibroblast growth factor 23 (cFGF23) and: (a) plasma phosphate, P, (b) plasma parathyroid hormone, PTH, and (c) plasma calcitriol, CTR, concentrations. Table S1: Sequences of the primers used for real-time RT-PCR. Table S2: Food, calcium (Ca) and phosphorus (P) intake in the six experimental groups at the end of the experiment.
