# Supplementary material for: Oral Acid Load Down-Regulates Fibroblast Growth Factor 23
Source: Nutrients. 2022 Feb 28;14(5):1041. doi: 10.3390/nu14051041 (PMC8912769; doi:10.3390/nu14051041)
Supplement: Supplementary file 1 [file nutrients-14-01041-s001.zip › Table S1_Supplementary.pdf]

Table S1. Sequences of the primers used for real-time RT-PCR.

Gene	Forward primer (5′-3′)	Reverse primer (5′-3′)
FGF23	TGGCCATGTAGACGGAACAC	GGCCCCTATTATCACTACGGAG
Tbp	ACTCCTGCCACACCAGCC	GGTCAAGTTTACAGCCAAGATTCA

All primers were purchased from Eurofins Genomics Germany GmbH, Ebersberg, Germany.
FGF23, fibroblast growth factor 23; Tbp, TATA sequence binding protein.
