# Supplementary material for: Oral Acid Load Down-Regulates Fibroblast Growth Factor 23
Source: Nutrients. 2022 Feb 28;14(5):1041. doi: 10.3390/nu14051041 (PMC8912769; doi:10.3390/nu14051041)
Supplement: Supplementary file 1 [file nutrients-14-01041-s001.zip › Table S2_Supplementary.pdf]

Table S2. Food, calcium (Ca) and phosphorus (P) intake in the 6 experimental groups at the end of the experiment.

	Food intake (g/day)	Ca intake (mg/day)	P intake (mg/day)
Control	16.63 ± 0.48	99.78 ± 2.86	99.78 ± 2.86
Control+Acid	16.11 ± 0.23	96.65 ± 1.41	96.65 ± 1.41
1/2 Nx	11.69 ± 0.47[a]	70.13 ± 2.81[a]	70.13 ± 2.81[a]
1/2 Nx+Acid	11.26 ± 0.41[a]	67.59 ± 2.46[a]	67.59 ± 2.46[a]
5/6 Nx	13.99 ± 0.31[a]	83.93 ± 1.89[a]	167.9 ± 3.78[a]
5/6 Nx+Acid	8.49 ± 0.83[a]*	50.94 ± 4.98[a]*	101.9 ± 9.98*

Letters ([a]) indicate significant differences (*P*<0.05) vs control group. Asterisks (*) indicate differences vs its non-acidotic counterpart (*P*<0.05). Values are means ± SE.
