# Supplementary figures and images for: Oral Acid Load Down-Regulates Fibroblast Growth Factor 23
Source: Nutrients. 2022 Feb 28;14(5):1041. doi: 10.3390/nu14051041 (PMC8912769; doi:10.3390/nu14051041)

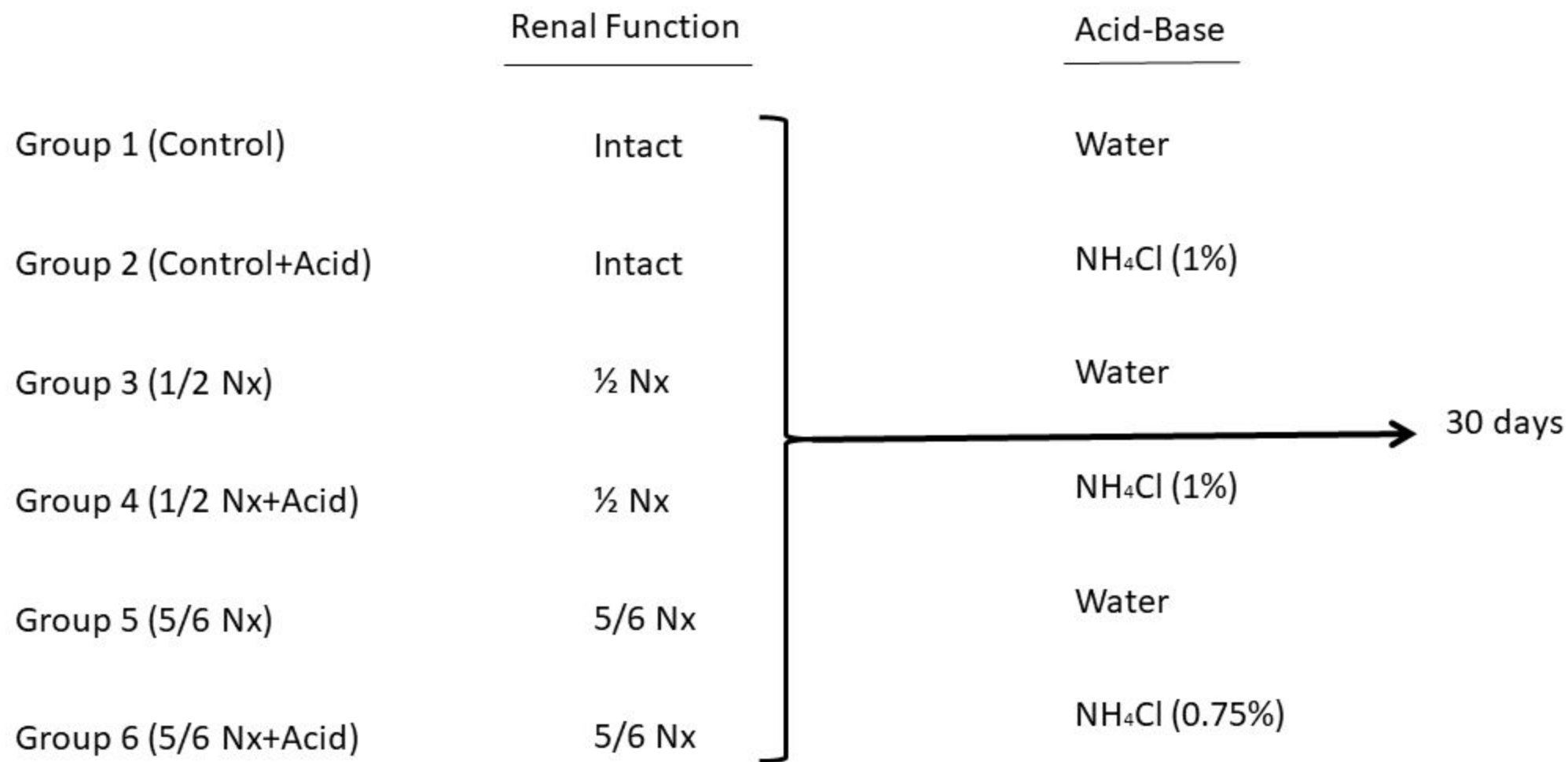

Supplement: Supplementary file 1 [file nutrients-14-01041-s001.zip › Figure S1.pdf]

a)

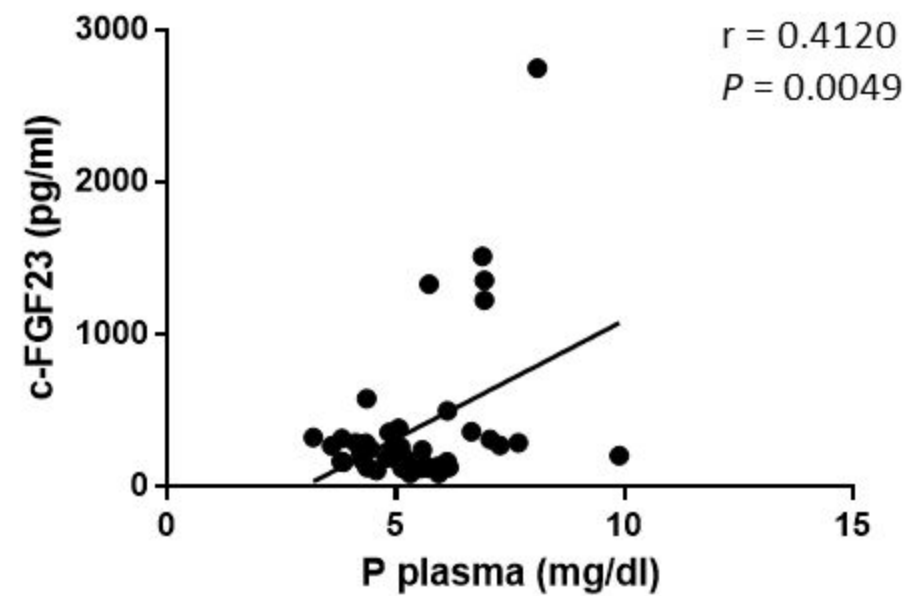

b)

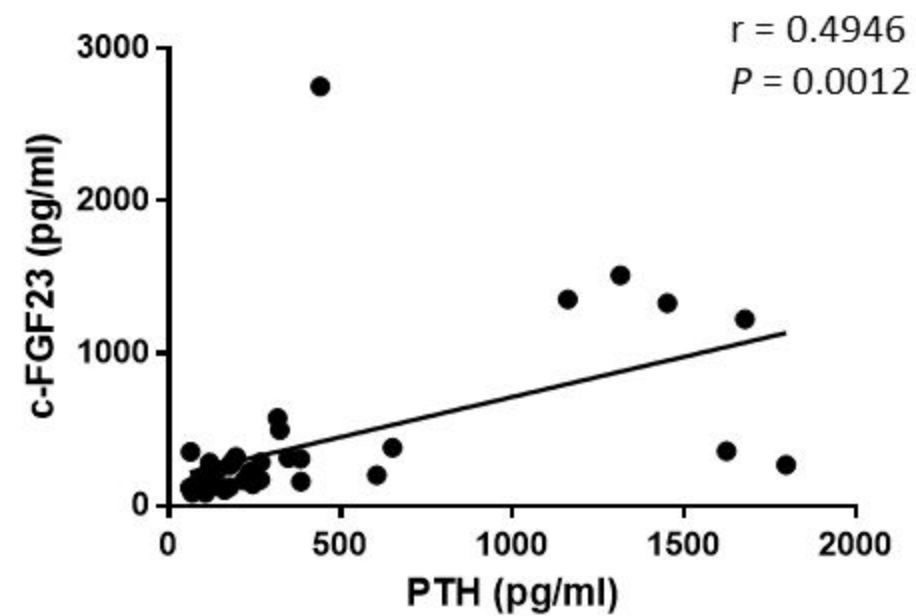

c)

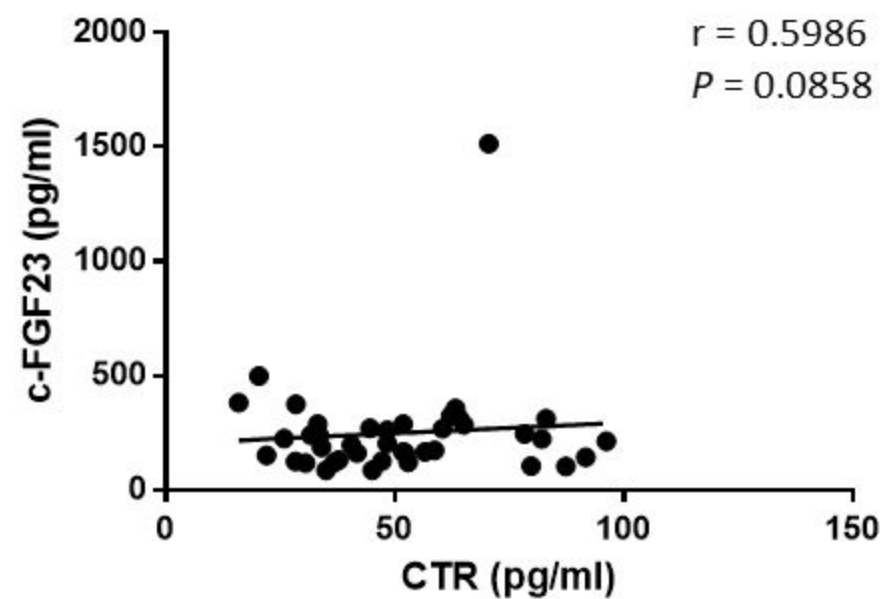

Supplement: Supplementary file 1 [file nutrients-14-01041-s001.zip › Figure S2.pdf]
